# Improving the Completion of Mental Capacity Act (MCA) Assessments and Deprivation of Liberty Safeguards (DoLS) in Complex Medical Units at the John Radcliffe Hospital

**DOI:** 10.7759/cureus.94536

**Published:** 2025-10-14

**Authors:** Soundarya Soundararajan, Alice Hindmarsh

**Affiliations:** 1 Internal Medicine, Broomfield Hospital, Chelmsford, GBR; 2 Internal Medicine, Southmead Hosptial, Bristol, GBR

**Keywords:** acute delirium, cognitive disorders, dementia care, deprivation of liberty safeguards, geriatrics population, uk mental capacity act 2005

## Abstract

Objective

Cognitive disorders can impair decision-making ability in older adults. In patients with cognitive disorders, including cognitive impairment, dementia and delirium, a mental capacity assessment (MCA) should be undertaken to assess whether a patient can consent to inpatient treatment. If a patient is found not to have capacity, a Deprivation of Liberty Safeguards (DoLS) should be authorised. This audit evaluates whether MCA and DoLS are used appropriately across the four Complex Medical Units (CMUs), which treat multimorbid patients, at the John Radcliffe Hospital.

Methods

The first and second rounds of assessment were completed on September 26, 2022 (n=65) and December 13, 2022 (n=66), respectively. Inpatients in CMUs aged ≥70 years were assessed for records of Abbreviated Mental Test Score (AMTS) <8, or diagnosis of delirium or cognitive impairment, which may indicate a lack of capacity to consent to inpatient admission. Where these criteria were met, it was assessed whether patients had a mental capacity assessment regarding hospital admission and a DoLS application if found to lack capacity.

Results

Patient characteristics were similar across the two cycles. In the first cycle, 66.2% (n=43) had AMTS assessment completed. Of the 62 eligible patients, 27.4% had a mental capacity assessment and 17.7% had DoLS in place. Interventions included MCA-DoLS teaching to CMU doctors and a week-long pilot measure in CMU-B to discuss MCA-DoLS during daily board rounds. In the second cycle, 72.7% (n=48) had AMTS assessment completed. Of the 58 eligible patients, 25.9% had a mental capacity assessment and 12.1% had DoLS in place.

Conclusion

MCA and DoLS protect patient’s rights while delivering quality care. Our audit has identified gaps in the current practice. Though educating doctors is effective, further work, including educating the multidisciplinary team, could help achieve higher rates of MCA-DoLS completion.

## Introduction

Cognitive disorders, including dementia, cognitive impairment and delirium, are more prevalent in older people, who form the majority of the inpatient population [1,2]. There is an increased incidence of these cognitive disorders that might impair the ability of the patient to make informed decisions, and they may lack the capacity to do so [3,4]. Studies have suggested that up to half of the patients in general medical wards may have dementia [2] and delirium can affect up to 50% of patients in hospital over the age of 65 [3]. These cognitive disorders are common, serious and often go unrecogonised [3]. Therefore, the identification of cognitive disorders is necessary to provide holistic care. A 2015 study by Pendlebury et al. has shown that subjective complaints about cognition agree poorly with objective cognitive deficit [1]. Hence, routine cognitive screening is recommended for all older people who are admitted to the hospital. The 10-point Abbreviated Mental Test Score (AMTS) is widely used as a cognitive screening tool in hospitals. The UK guidelines for dementia screening recommends using an AMTS cut-off of <9 for referral to specialist assessment for possible dementia while a cut-off of <8 is widely cited in the literature [5].

The Mental Capacity Act (MCA) 2005 aims to protect and restore power to vulnerable people in the population who may lack capacity to make certain decisions [6-8]. The purpose of mental capacity assessment is to determine whether a person can make a decision. It relates to a particular decision at a particular time [6-8]. The five principles of MCA are [8]:

1. Assume that people have capacity.

2. People must be supported to make their own decisions.

3. People may make unwise decisions but not necessarily lack capacity.

4. Any decision made must be in a person’s best interests.

5. The least restrictive option should be chosen.

A person is deemed to have mental capacity regarding a particular decision when they can [8]:

1. Understand information related to that decision

2. Retain given information

3. Weigh up information as part of decision-making

4. Communicate the decision.

While the MCA allows some restrictions to be put in place, they can only be done when the restraints are in the person’s best interests and are the least restrictive option [8]. Additional safeguards in the form of Deprivation of Liberty Safeguards (DoLS) are legally required where restrictions and restraints are thought to deprive the person of their liberty [9]. DoLS were introduced as an amendment to the MCA and was implemented on April 1, 2009 [10]. The DoLS application process begins when a potential deprivation of liberty has occurred or is about to occur in a hospital or care home [10]. Care homes or hospitals complete an application form to seek authorisation for the deprivation and submit it to the local authority. The local authority reviews the application with a team based on the following six criteria [10]:

1. Age: the person must be aged ≥18 years old.

2. Mental capacity: the person should be assessed as lacking capacity to make a decision.

3. Mental health: the person should be assessed as having a mental health disorder.

4. No Refusals: the person must not have made a valid advance decision or have someone appointed who is in opposition to the proposed care.

5. Eligibility: the person is eligible unless they meet the criteria for detention under the Mental Health Act.

6. Best Interests: deprivation of liberty is occurring in the best interest of the individual.

DoLS application is granted where all the six criteria are met, which means the individual can be legally deprived of their liberty by the hospital or care home. A 2014 court judgment devised an ‘acid test’ to evaluate who should be under a DoLS [11]. It clarified that if a person lacks capacity, is under constant supervision and would not be allowed to leave, then they should be placed under a DoLS. This would apply to many patients in nursing bays (under constant supervision by nursing staff) within geriatric wards.

During daily reports across the four Complex Medical Units (CMU) at the John Radcliffe Hospital in Oxford, it was noted that patients who are deemed by the Trust to require a DoLS do not always have one, and those who may require one are not necessarily being assessed. These wards usually treat multi-morbid patients. Therefore, an audit was undertaken to improve the rates of appropriate assessment of mental capacity and authorisation of DoLS on the CMU wards.

## Materials and methods

The standard for the audit was that all patients over 70 should have an AMTS score within 72 hours of admission. In addition, all patients with a diagnosis of cognitive impairment, dementia, delirium or an AMTS<8 should have a capacity assessment (usually by medical staff) to ascertain whether or not they can consent to inpatient admission for treatment. If they cannot, then a DoLS should be completed (usually by nursing staff).

The data collection for this audit comprised two parts. Initially, it was evaluated how many patients had an AMTS score performed within the first 72 hours of admission. All patients over the age of 70 years old were included as patients who met the criteria, as set out by our standard. Data was collected for patients on one snapshot day across all four CMU wards (around 20 patients per ward). The first round of data collection happened in September 2022, and the second round in December 2022. The percentage of patients who had an AMTS score within 72 hours was calculated. More specifically, if an AMTS score was recorded as ‘not feasible’, we recorded the reason for this, including patient ‘too unwell’, ‘uncooperative’ or dysphasic’. The data was collected by searching the electronic patient record (EPR) for the proforma used to record AMTS scores.

In the second part of the data collection, how many eligible patients had an MCA or DoLS was assessed. All patients who were aged 70 and over with a diagnosis of cognitive impairment, dementia or delirium or an AMTS <8 were included as eligible patients, as set out by our standard. We collected data for patients on the same snapshot day as the first part of our data collection. The first round of data collection was done in September 2022 and the second round in December 2022.

Demographics of the eligible patients were collected, including age, sex, gender and length of admission. More detailed information was also collected, including diagnosis of cognitive disorders, delirium and other medical comorbidities. Then, details of MCAs were collected. This information was collected by searching for the specific proforma used on the EPR to record capacity assessments. If this had not been completed, we searched if capacity assessments had been recorded in the main body of the notes. The outcome of the MCAs was recorded as either the patient does or does not have capacity to consent to inpatient treatment. The percentage of patients who had an MCA performed among the patients who were deemed eligible was calculated.

If the patient was recorded as not having capacity, it was then assessed whether the patient had a DoLS in place. This was done by searching for the proforma used to record DoLS on EPR. If this was not present, we searched for a record of DoLS in the main body of the notes. The percentage of patients who had a DoLS in place in the patients deemed not to have capacity was calculated.

All data was recorded in a Microsoft Excel (Microsoft, Redmond, WA) spreadsheet. Continuous variables were presented as means and compared using unpaired (Student’s) t-test, to evaluate differences in the patient population included in the first and second rounds of the audit. Categorical variables were compared using Fisher’s exact test. A probability value of <0.05 was considered to be statistically significant. All the used measurements, scales and scoring systems were free to use and do not require permission or a licence.

## Results

Initial results

The initial data collection of the audit comprised 74 patients in total. Of those, 65 patients across the four CMUs were included in the audit. The remaining nine patients were not included in the study as they were younger than 70 years. Of the 65 patients, there were 31 female and 34 male patients. The mean age of the patient sample included in the audit was 85.1 years. The mean length of hospital stay was 14.5 days. This included stay in the Emergency Department (ED), Emergency Assessment Unit (EAU), short-stay wards and CMU.

**Figure 1:**
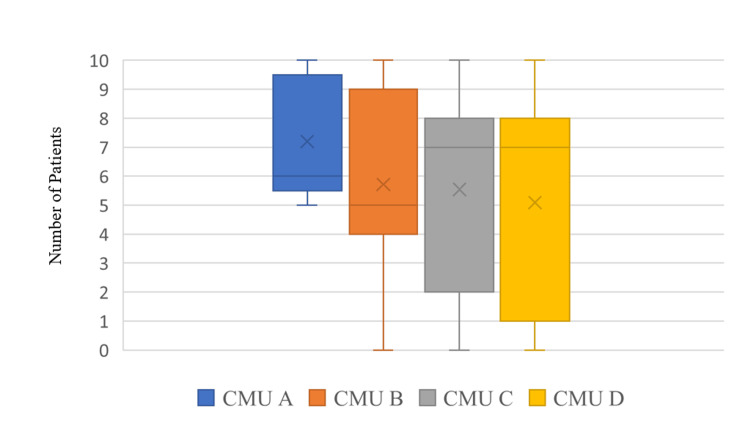
Distribution of AMTS scores within 72 hours of admission across the four CMUs Source: AMTS score [5].

Of the 65 patients, AMTS assessment was performed within 72 hours of admission in 43 patients (66.2%). Very often, the initial AMTS assessment is performed in ED or EAU where the patient is first clerked by the medical team. Where an AMTS was performed, either the score or the reason why the assessment was not feasible was recorded. AMTS score was recorded in 34 patients in total (52.3%). Where AMTS was recorded as ‘Not feasible’ (nine patients, 13.8%), common reasons cited include ‘uncooperative’, ‘too unwell’ or ‘dysphasic’. Twenty-two patients (33.8%) did not have an AMTS assessment within 72 hours of admission and 15 patients (23.1%) never had an AMTS assessment during their hospital stay. Analysing the AMTS scores, 24 patients (36.9%) had an AMTS <8. The AMTS score distribution across the four CMUs is shown in Figure 1.

**Figure 2:**
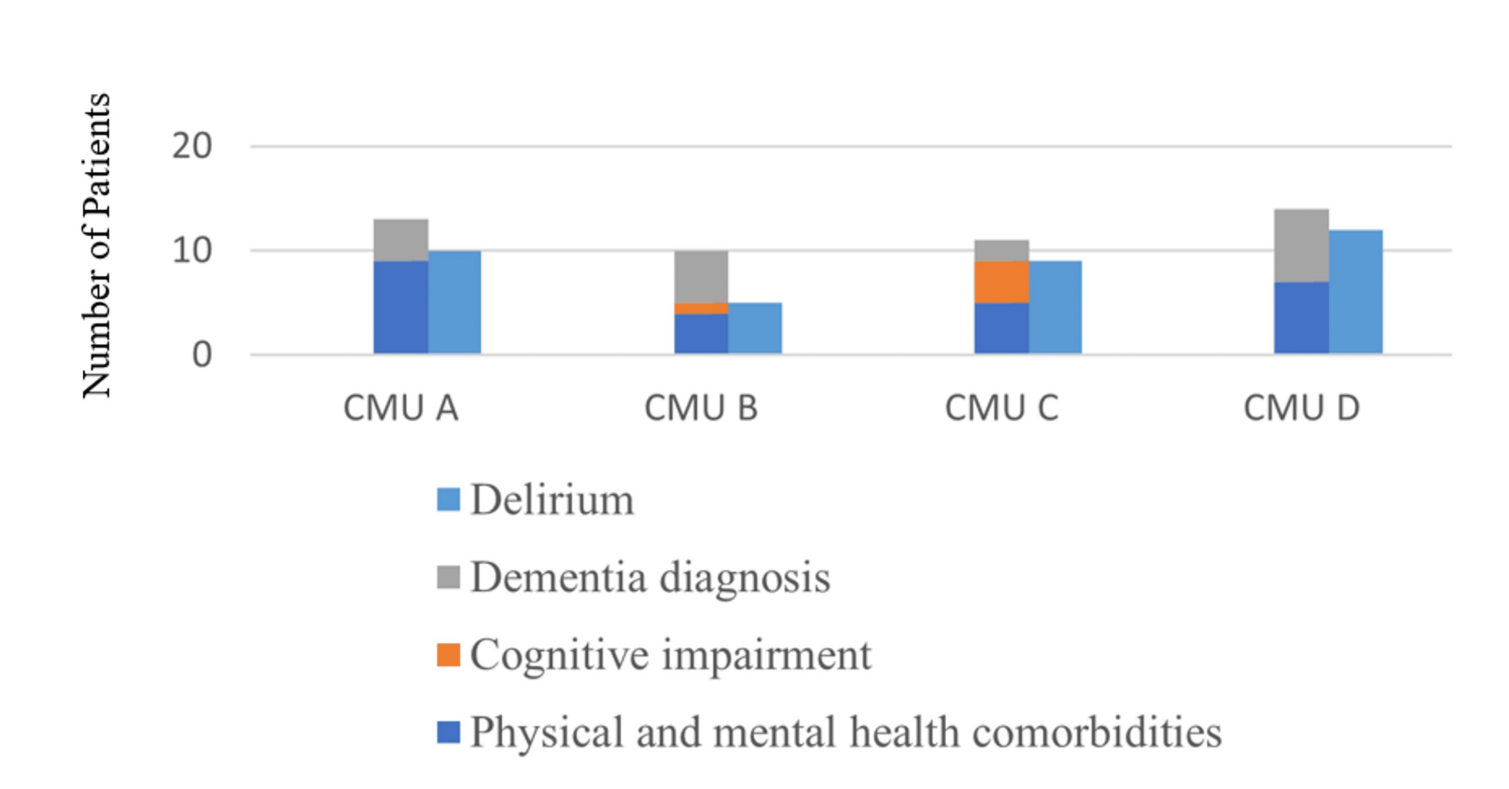
Relevant medical history suggestive of impairment of the mind or brain

Apart from the AMTS, the past medical history was also reviewed. Forty-eight out of 65 patients (73.8%) had a relevant medical history as shown in Figure 2.

Though a total of 36 patients (55.4%) had a diagnosis of delirium, 25 of those also had a diagnosis of physical and mental comorbidities, cognitive impairment or dementia. In total, 62 patients in the audit sample would qualify for a mental capacity assessment and set out in our criteria. 

**Figure 3:**
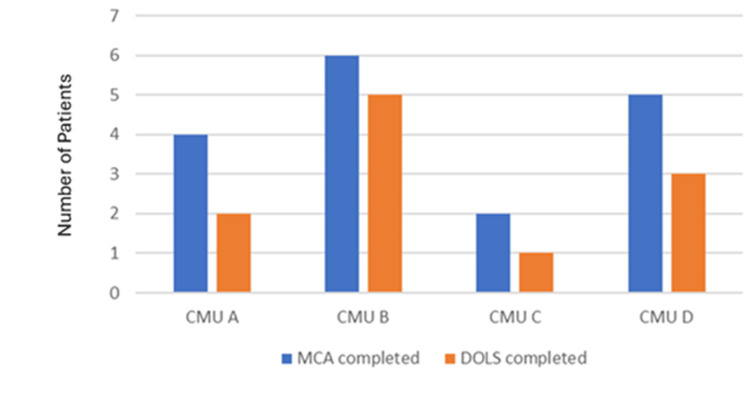
Record of mental capacity assessment (MCA) and Deprivation of Liberty Safeguards (DoLS) in first round of data collection Source: MCA [6] and DoLS [9].

Mental capacity was assessed and documented in only 17 patients (27.4%). Among the 17 patients who were deemed not to have capacity to consent to hospital admission, DoLS was only completed in 11 patients (17.7%) as shown in Figure 3.

The initial data collection identified that there were gaps in the assessment and record of the mental capacity of patients as well as DoLS authorisation.

Change implementation

Following our initial data analysis, we presented the data during the Gerantology lunchtime teaching for doctors in October 2022, together with an educational update on MCA and DoLS. In addition, we piloted another measure in one of the wards, CMU-B, for a week where MCA and DoLS were discussed as a mandatory topic during board rounds. Board rounds are attended by a multi-disciplinary team including doctors, nurses, therapists and discharge coordinators.

Results after the implementation of change

The second round of data collection was performed to evaluate the effectiveness of the changes in December 2022 using the same methodology.

There were 70 patients in total in this round of data collection. Of those, 66 patients across the four CMUs were included in the audit. The remaining four patients were not included in the study as they were younger than 70 years. Of the 66 patients, there were 34 female and 32 male patients. The mean age of the patient population included in the audit was 84.7 years. The mean length of stay was 12.1 days.

Of the 66 patients, an AMTS assessment was performed within 72 hours of admission in 48 patients (72.7%). Of the 48 patients who had an AMTS assessment within 72 hours of admission in the second cycle of the audit, an AMTS score was recorded in 33 patients and AMTS was ‘not feasible’ in 15 patients. Twenty-one patients scored <9 (32.3%). Furthermore, 43 out of 66 patients (65.1%) had a relevant medical history suggestive of impairment of the mind or brain. Though a total of 36 patients (54.5%) had a diagnosis of delirium, 31 of those also had a diagnosis of physical and mental comorbidities, cognitive impairment or dementia.

**Figure 4:**
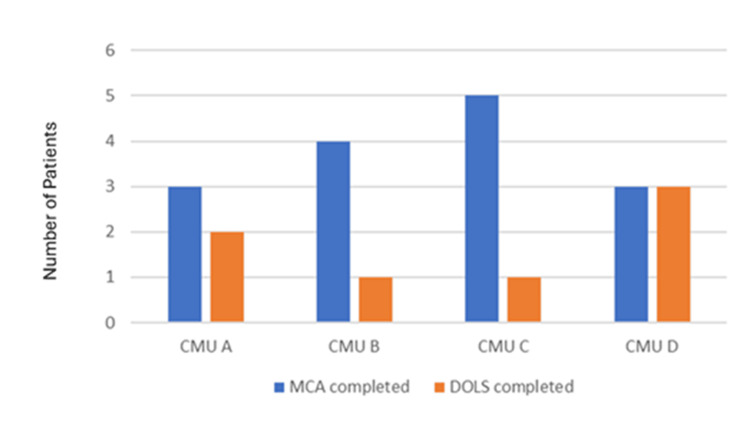
Record of mental capacity assessment (MCA) and Deprivation of Liberty Safeguards (DoLS) in the second round of data collection Source: MCA [6] and DoLS [9].

In total, 58 patients (87.8%) would qualify for a mental capacity assessment. Mental capacity was assessed and documented in 15 patients (25.9%) while DoLS was completed in seven patients (12.1%) as shown in Figure 4.

Looking specifically at the data for CMU-B, where MCA-DoLS was discussed at the daily board round, 11 patients qualified for a mental capacity assessment in the second cycle of the audit. Mental capacity was assessed and documented in 36.3% of the patients as opposed to 50% in the first cycle of the audit. Of those who were assessed not to have the capacity to consent to hospital admission, a lower proportion of patients had DoLS authorisation in the second cycle compared to the first cycle of the audit.

## Discussion

This audit was presented as an oral presentation at the European Geriatric Society Conference in Helsinki, 2023. The audit cycle comprised patients with similar baseline characteristics. There was no statistically significant difference in age (95% confidence interval (CI): -1.96 to 2.79, p=0.73 using Student’s t-test) and length of stay (95% CI: -1.50 to 6.43, p=0.22 using Student’s t-test).

Cognitive disorders are common among older adults and are associated with increased morbidity and mortality [12]. Given the ever-growing elderly population, it is important to have a simple and effective tool to identify cognitive impairment in patients. The AMTS, which comprises a 10-point questionnaire, is thought to be applicable to the majority of older adults currently [5,13]. In the second cycle of data collection, 72.7% of patients had an AMTS assessment within 72 hours of admission, as opposed to 66.2% in the first cycle (p=0.45 using Fisher’s exact test). Although the absolute number of AMTS assessments has increased, the increase was not statistically significant.

In contrast, of all the patients who would qualify for mental capacity assessment, only a minority had an assessment recorded on the EPR in both data collections. The difference in the completion of mental capacity assessment between the first and the second data collection was not statistically significant (p=1.0 using Fisher’s exact test). Similarly, the difference in the completion of DoLS between the first and the second data collection was not statistically significant (p=0.48 using Fisher’s exact test). Furthermore, the proportion of completion of DoLS, in comparison with the number of patients deemed to lack capacity based on the mental capacity assessment, was lower than in the first cycle of the audit. Consistent with the overall findings, the proportion of completion of DoLS was lower in the second cycle than in the first cycle of the audit in CMU-B, where a separate pilot measure was rolled out.

The audit highlighted that there is a discrepancy between the number of patients having MCA assessment completed and deemed not to have capacity and the number of DoLS being completed. One reason for the discrepancy could be the lack of communication between the medical staff who complete the MCA assessment and the nursing staff who complete the DoLS application. The National Health Service (NHS) Digital official statistics on DoLS for the period April 1, 2022 to March 31, 2023 revealed an increase of 11% in the number of DoLS notifications from hospitals compared to the previous year, which was found to be closer to the rate of growth in applications before the COVID-19 pandemic [10]. The number of DoLS notifications from adult social care services and hospitals was the lowest during the beginning of the COVID-19 pandemic, when attention shifted toward infection prevention and control [10]. As noted by the Care Quality Commission (CQC) in the 2020/2021 edition of State of Care, there is variation in the knowledge and understanding of the DoLS legislation and quality of training around the topic [14]. This suggests that hospitals may not effectively escalate DoLS applications when needed.

As part of ‘change implementation’, all Geriatric Medicine trainees working in the CMUs had teaching on MCA and DoLS. The teaching aimed to provide a theoretical understanding of the principles of the Mental Capacity Act. The low MCA-DoLS completion rate suggests that further training is needed to go beyond learning the principles of the assessments, to application in clinical practice. This finding was also noted by Hinsliff-Smith et al. [15]. The difficulties of translating the principles into clinical practice can be influenced by individual and system factors. Looking at individual factors, the second cycle was conducted soon after some junior doctors had changed rotations, which suggests that they may not have known about the MCA-DoLS roll-out. Looking at system factors, the second cycle of the audit was conducted during one of the busiest times of the year, when hospital teams were faced with high patient turnover. Time pressures may have limited the ability of doctors and nurses, respectively, to complete the paperwork associated with the mental capacity assessment and DoLS authorisation.

Future work

Following our second data analysis, we aim to include a section in the CMU Admission Proforma that includes ‘AMTS outcome, relevant medical history suggestive of impairment of the mind or the brain and MCA-DoLS’. We believe this would serve as a reminder for the medical team to consider mental capacity assessment during the daily ward rounds.

With rotating junior doctors, it is important to have regular education and updates for trainees often completing MCA assessments. However, MCA assessments do not need expert assessments and can be completed by any individual caring for someone who may lack capacity [16]. Though educating the medical team has shown some success, it is equally important to include the multi-disciplinary team (MDT) in the MCA-DoLS training, as every member has a role to play in the provision of holistic and high-quality care for older people. Raising awareness about the importance of capacity assessment and DoLS through posters around the wards would encourage the initiation of related discussions and reduce the likelihood of not completing the necessary forms when appropriate. We have also shared our data with the Safeguarding Lead for DoLS and the Matron for the CMU wards, who are implementing further nurse education. Further work on communication between healthcare professionals is also needed to ensure DoLS applications are completed when an MCA assessment deems a patient to lack capacity.

In addition, the assessment of mental capacity and consideration of the requirement for DoLS is relevant to other clinical areas. As such, the work will be presented at Acute Medicine Clinical Governance. Valuable information can also be obtained by comparing rates of MCA and DoLS completion in other hospital sites of the Oxford University Hospitals Trust.

Lastly, the CQC report also highlighted that there are delays and backlogs at the local authority level [14]. It is expected that Liberty Protection of Safeguards will replace DoLS in the near future [14]. Following this, every Trust, rather than the local authority, will be responsible for the completion of paperwork and, therefore, possibly more open to scrutiny.

Limitations of the study

There are some limitations to this study. Firstly, the audit was only completed on the CMU wards at the John Radcliffe Hospital, but older patients are admitted across most other wards in the hospital. Therefore, we are not capturing the whole cohort of eligible patients within the hospital. Furthermore, we simultaneously performed our interventions of change - the teaching session and board round initiative - resulting in ambiguity about which intervention had the most impact. Finally, the teaching session given was on one afternoon and subsequently not all of the doctors working within the Gerontology department will have been present and therefore may have been unaware of the initiative being undertaken.

## Conclusions

The implementation of MCA and DoLS helps to ensure that patient’s who lack capacity to make decisions receive holistic care. Our audit evaluated the completion of MCA and DoLS in the four CMUs of the John Radcliffe Hospital and has highlighted that there are gaps in the current performance, including in the identification of cognitive disorders and in applying the MCA and DoLS frameworks. Though education of the medical team has shown effectiveness, further work is needed to achieve a higher rate of completion. In particular, education of the MDT is also needed to maximise achievement.
